# Silver-Decorated and Silica-Capped Magnetite Nanoparticles with Effective Antibacterial Activity and Reusability

**DOI:** 10.1021/acsabm.3c00122

**Published:** 2023-06-05

**Authors:** Shadab Dabagh, Somayeh Asadi Haris, Behzad Khatamsaz Isfahani, Yavuz Nuri Ertas

**Affiliations:** † ERNAM—Nanotechnology Research and Application Center, Erciyes University, Kayseri 38039, Türkiye; ‡ Department of Biotechnology, Faculty of Advanced Sciences and Technologies, University of Isfahan, Isfahan 81746-73441, Iran; § Department of Biomedical Engineering, Erciyes University, Kayseri 38039, Türkiye

**Keywords:** green synthesis, magnetite, silver, nanoparticles, antibacterial, magnetic

## Abstract

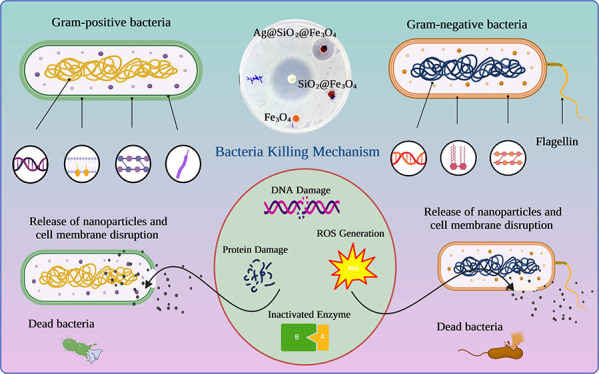

Fruits are safe, toxin-free, and biomolecule-rich raw materials that may be utilized to decrease metal ions and stabilize nanoparticles. Here, we demonstrate the green synthesis of magnetite nanoparticles which were first capped with a layer of silica, followed by the decoration of silver nanoparticles, termed Ag@SiO_2_@Fe_3_O_4_, by using lemon fruit extract as the reducing agent in a size range of ∼90 nm. The effect of the green stabilizer on the characteristics of nanoparticles was examined via different spectroscopy techniques, and the elemental composition of the multilayer-coated structures was verified. The saturation magnetization of bare Fe_3_O_4_ nanoparticles at room temperature was recorded as 78.5 emu/g, whereas it decreased to 56.4 and 43.8 emu/g for silica coating and subsequent decoration with silver nanoparticles. All nanoparticles displayed superparamagnetic behavior with almost zero coercivity. While magnetization decreased with further coating processes, the specific surface area increased with silica coating from 67 to 180 m^2^ g^–1^ and decreased after the addition of silver and reached 98 m^2^ g^–1^, which can be explained by the organization of silver nanoparticles in an island-like model. Zeta potential values also decreased from −18 to −34 mV with coating, indicating an enhanced stabilization effect of the addition of silica and silver. The antibacterial tests against *Escherichia coli* (*E. coli*) and *Staphylococcus aureus* (*S. aureus*) revealed that the bare Fe_3_O_4_ and SiO_2_@Fe_3_O_4_ did not show sufficient effect, while Ag@SiO_2_@Fe_3_O_4_, even at low concentrations (≤ 200 μg/mL), displayed high antibacterial activity due to the existence of silver atoms on the surface of nanoparticles. Furthermore, the in vitro cytotoxicity assay revealed that Ag@SiO_2_@Fe_3_O_4_ nanoparticles were not toxic to HSF-1184 cells at 200 μg/mL concentration. Antibacterial activity during consecutive magnetic separation and recycling steps was also investigated, and nanoparticles offered a high antibacterial effect for more than 10 cycles of recycling, making them potentially useful in biomedical fields.

## 1 Introduction

Nanoparticles (NPs) are distinguished from bulk materials by their unique electrochemical, optical, and thermal characteristics, as well as their much larger surface area to volume ratio. Due to their distinctive features, NPs have found broad usage in a variety of scientific areas, including agriculture, biotechnology, chemistry, communications, electronics, energy, material science, medicine, microbiology, optics, and various engineering fields.^1−7^ From metals and their related oxides, NPs have been created using several physical and chemical procedures, including ultrasonication, microwave irradiation, laser vaporization, wet impregnation, and sol–gel approaches.^8,9^ These synthesis methods intensively use toxic and hazardous substances that harm the environment and require stringent conditions and expensive equipment to operate.^10,11^ Therefore, the development of proficient, economical, and environmentally friendly green approaches for the synthesis of NPs is of high value. Plant extracts can serve as a viable precursor for the environmentally friendly synthesis of nanomaterials.^12^ In comparison to conventional methods, the preparation of NPs using green chemistry techniques has a number of advantages, such as low cost, environmental friendliness, safe handling, and, at the source level, stabilizing materials that are derived from plants, microorganisms, or other natural resources to minimize health and environmental concerns.^13^

Different types of fruits, bacteria, actinomycetes, fungi, yeast, viruses, and other microbes can be used to produce stable and functionalized NPs.^14^ Among all types of reducing biomolecules, plant extracts have received more attention because they are simple to make and can be scaled up for NP production on an industrial scale.^15^ Plant-produced NPs are more stable, and their rate of synthesis is quicker than that of microorganisms. In addition, the NPs have a greater variety of sizes and shapes than those created by other biomolecules. The advantages of employing plants and plant-derived materials for metal NP biogenesis have aroused the interest of researchers, who are investigating techniques of metal ion absorption and bioreduction by plants, as well as the likely process of metal NP production in plants.

Fruits of edible plants are non-toxic, biomolecule-rich source materials that decrease metal ions and stabilize NPs. Numerous biomolecules derived from plant sources may function as reducing or capping agents. This study focuses on lemon, a popular fruit rich in antioxidants such as polyphenols, limonoids, citric acid, ascorbic acid, and vitamins that have the ability to decrease ions in high oxidation states. As capping agents, some phytochemicals, such as proteins and carbohydrates containing ionic groups, can function. In spite of this, NP production and stabilization in aqueous media can use lemon fruit extract.^16^

Magnetic NPs that possess superparamagnetic characteristics along with low toxicity and biocompatibility have the potential to be used in a variety of technical applications. Magnetic storage, magnetic ink printing, microwave absorption, biosensors, bioseparation, in vivo drug administration, immunomagnetic arrays, magnetic resonance imaging contrast agents, hyperthermia therapy for cancer, and antibacterial agents are a few examples of these uses.^17−21^ Due to desired magnetic characteristics such as easy separation under external magnetic fields and high biocompatibility, magnetite (Fe_3_O_4_) NPs have been the most investigated type of magnetic material for biomedical applications. They display a distinctive magnetism, known as superparamagnetic, where the NPs have the ability to behave like atomic paramagnets and act as a single magnetic domain. Unlike other metal oxide NPs or composites, these NPs display substantial saturation magnetization, high field irreversibility, and magnetic anisotropy. Bare Fe_3_O_4_ NPs display high chemical activity and easily oxidize in atmospheric air, which results in the loss of magnetic properties. Iron oxide NPs may be stabilized in colloidal solutions and biological fluids when their surfaces are functionalized and coated.^22^ A wide variety of polymers and inorganic materials are utilized as coating agents, which provide a template that controls particle growth and nucleation while maintaining the size, shape, and colloidal stability of magnetite NPs. In addition, these templates produce a confinement effect that prohibits physical contact between the generated particles in colloidal solution and also hinders immunogenic attack in biological fluids, a phenomenon known as the ″stealth effect″. In addition, the coating layer inhibits the degradation of magnetic core materials in biological media and permits the regulated release of pharmaceuticals based on the coating material, its weight, and loading capacity.

A non-toxic coating material is silica, which inhibits the superparamagnetic core from aggregating in liquid media and enhances the stability, biodegradability, and biocompatibility of NPs while reducing their toxicity. Besides, the silica layer generates additional silanol groups that easily react with other compounds. Numerous techniques, including the sol–gel process,^23^ aerosol pyrolysis,^24^ microemulsion,^25^ and the Stöber method,^26^ have been proposed for coating the surface of NPs with silica. The Stöber method can be used to passivate the magnetite NP surface with silica without using any surfactants.^27^

Precious metal NPs (such as Au, Pt, and Ag) have gained significant scientific attention recently because of their antibacterial activity and superior physicochemical characteristics.^5,28^ Specifically, silver NPs have been the subject of substantial research on eliminating bacteria. Silver NPs have a tendency to aggregate, change form, and disrupt the surface state due to their high surface energy and van der Waals interactions, which may result in the loss of their intrinsic activity and selectivity.^29^ Silver NPs can be attached to a variety of support materials, including polymer nanospheres, magnetic materials, and carbon materials. Functionalizing magnetite NPs with silica can leverage the potential of each material because silica can be easily immobilized, and magnetic recycling of NPs would be possible for repeated usage.^28^

Solvothermal reaction,^30^ electroless-deposition method,^31^ in situ wet chemistry,^32^ sonication technique^33^ and Stöber process^34^ have been used to produce silver-magnetite NPs. However, these methods have a variety of disadvantages, including a high cost of operation, challenges in controlling particle size, morphology, and phase composition, and the need for an additional step if a surfactant is utilized in the synthesis process. So, the green extraction and precipitation method has been used in this study so that fewer harmful chemicals are consumed, the process is more affordable, and high-energy processes like calcination are avoided.

In contrast to traditional synthesis strategies that offer low stability and employ harmful substances, here we report an efficient, easy, green, and economical technique for the preparation of Ag@SiO_2_@Fe_3_O_4_ NPs as a magnetic antibacterial agent. In order to prevent silver from aggregating and demonstrate a regulated release of silver ions, silica coating has been widely used as an ideal carrying material. This leads to long-term efficacy while reducing unwanted toxicities. The green synthesis strategy used no surfactants, chemical reducing agents, or capping agents, was performed at a low temperature, and does not need the prior synthesis of silver. The recyclable green synthesis NPs were also evaluated against two important foodborne pathogens (*Escherichia coli* and *Staphylococcus aureus*), where high antibacterial efficiency and low cytotoxicity were observed, which makes these NPs safe, affordable, and sustainable for antimicrobial and biomedical applications. The reusability of the NPs was also evaluated, and the results show that the prepared NPs can be used as a highly effective and reusable antibacterial agent in public health sectors with no considerable loss of performance.

## 2 Materials and Methods

### 2.1 Lemon Fruit Extract

**Figure 1:**
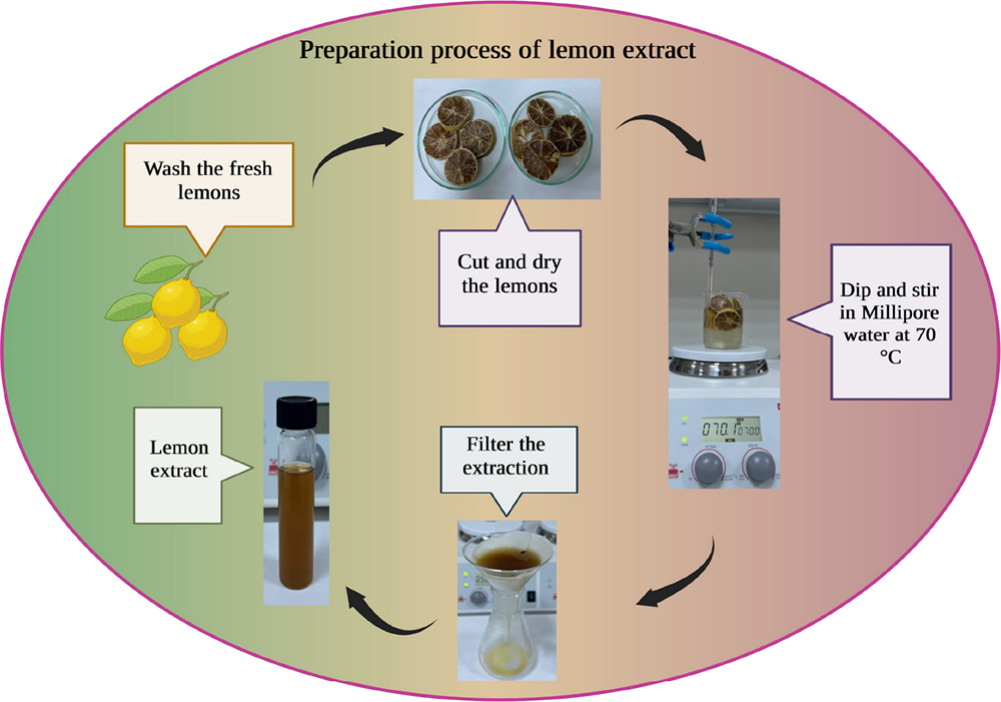
Schematic diagram of lemon extract preparation using the fresh lemon which was washed, cut, and dried at 60 °C. The dried lemon pieces were dipped in Millipore water and stirred at 70 °C; lemon extract can be used after filtration of the solution.

Fresh lemon (*Citrus limon*) fruits from the local market were washed with tap water and then with Millipore water, sliced, and dried at 60 °C for 10 h. Dry lemon pieces were mixed with Millipore water (20 g per 100 mL) and then stirred continuously at 100 rpm at 70 °C for 4 h. After the suspension had cooled to room temperature, filter paper was used to filter it. The extract was utilized for NP manufacturing and kept at −80 °C. The schematic diagram for the lemon extraction process is shown in Figure 1.

### 2.2 Green Synthesis of Fe_3_O_4_ Nanoparticles

FeCl_3_·6H_2_O (4.8 g, 0.018 mol, analytical grade, purity ≥98%, Sinopharm, China) and FeCl_2_·4H_2_O (1.8 g, 0.0089 mol, analytical grade, purity≥98, Sinopharm, China) were added to 100 mL of deionized water and stirred at 700 rpm until the salts completely dissolved. 8 mL of lemon extract was added to the solution, and then NaOH (1 M) was added to the solution until pH reached 11. The brown-black precipitate was separated by an external magnet, centrifuged, and washed five times with double-distilled water after the dispersion was transported to an autoclave lined with Teflon and heated to 180 °C for 8 h. Finally, it was vacuum-dried overnight at 70 °C.

### 2.3 Silica Coating of Fe_3_O_4_ Nanoparticles (SiO_2_@Fe_3_O_4_)

Silica coating of Fe_3_O_4_ NPs using a green method minimizes the complexity, cost, and hazardous chemicals required for the reduction of Ag^+^ ions, making the synthesis process cost-effective, non-toxic, and eco-friendly. So, using a modified Stöber process, NPs were coated with silica. The hydrolysis of tetraethyl orthosilicate (TEOS) in the presence of magnetite NPs was used. 0.1 g of produced magnetite NPs was dispersed in 50 mL of distilled water for 30 min using an ultrasonic water bath, and then the pH of the solution was raised to 10 by using aqueous ammonia. Later, 3 mL of TEOS was added drop by drop while shaking the solution at room temperature, and the solution was stirred overnight. The precipitates of the product were separated by an external permanent magnet and washed many times with ethanol and water (1:10). Finally, it was baked in a vacuum oven to yield NPs.

### 2.4 Green Synthesis of Silver Nanoparticles and Deposition onto SiO_2_@Fe_3_O_4_

**Figure 2:**
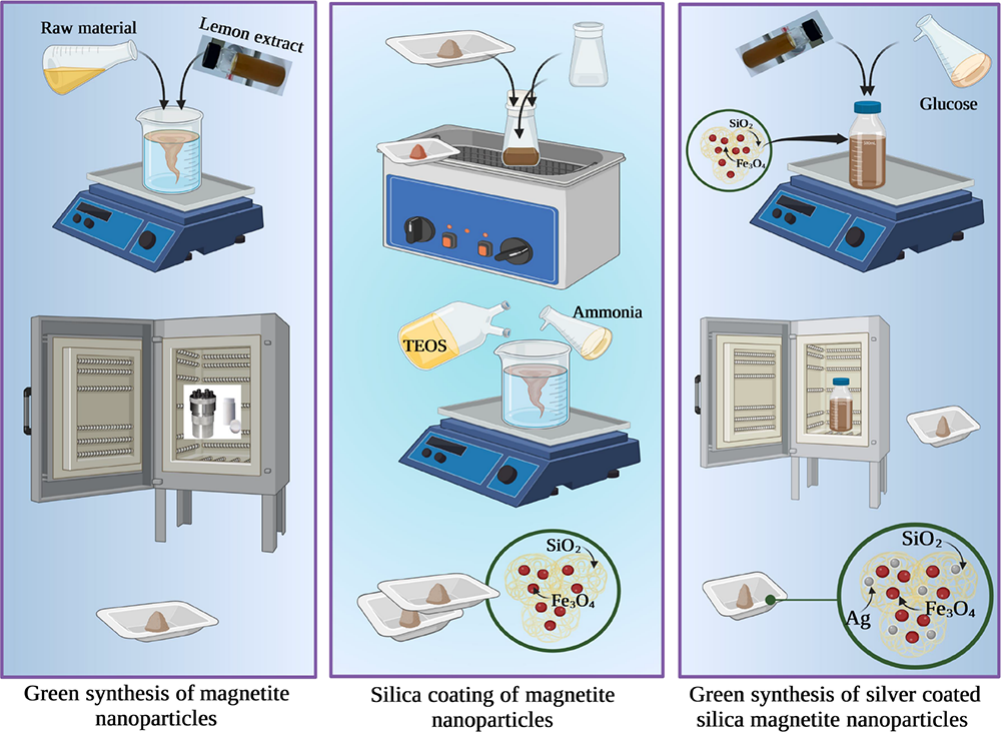
Schematic diagram of the Ag@SiO_2_@ Fe_3_O_4_ NPs: the first step is the green synthesis of Fe_3_O_4_ using lemon extract as the reducing agent. Stöber method was used as the surfactant-free method for coating the magnetite NPs with silica. Glucose and lemon extract as the stabilizer and reducing agent were used for the decoration of silver on SiO_2_@Fe_3_O_4_ NPs.

For the synthesis of silver NPs, the previously reported process of green synthesis was modified.^35^ Saxena et al. optimized various reaction parameters such as medium, AgNO_3_ concentration, reducing agent, pH, and temperature to maximize the generation of silver NPs that offer better antibacterial activity.^36^ Increasing the concentration of AgNO_3_ to 2 mM resulted in a complete reduction of Ag^+^. Also, the role of a green reducing agent in the formation of silver NPs was investigated using varying amounts of the reducing agent. The generation of Ag^+^ increases as the amount of the reducing agent increases. This could be due to a direct proportionate relationship between the amount of green synthesis agent and its release, which is responsible for the synthesis of silver NPs. The maximum production of silver NPs was achieved at pH 11–12, as evidenced by a change in color; similar findings were reported by other researchers as well.^37^ The effect of temperature on silver NP production was evaluated, with the maximum generation of Ag NPs observed at ∼80 °C. Based on these optimization protocols, 20 mL of citrus lemon extract was combined with 100 mL of 0.02 mol silver nitrate (AgNO_3_, Sigma Aldrich, USA) solution and stirred continuously at a speed of 300 rpm and at a temperature of 80 °C under the dark condition. The color of the colloidal suspension changed from yellow to brown after 1 h, and the mixed solution became reddish-brown, indicating the creation of silver NPs. AgNO_3_ (0.02 g) solution was added to a well-dispersed SiO_2_@Fe_3_O_4_ NPs (0.1 g) in deionized water, and then 0.1 mL of aqueous ammonia (25%, w/w) was added, where the Ag (NH_3_)^2+^ complex was formed, and 10 mL of citrus lemon extract was added as a stabilizer. Finally, a solution of glucose (C_6_H_12_O_6_, Merck, Germany) was introduced as the reducing agent, and the whole mixture was heated at 70 °C to accelerate the reduction reaction. With the addition of NaOH solution, the pH of the suspension was adjusted to 12, and the mixture was stirred for 45 min. The suspension was centrifuged three times at 12,000 rpm for 20 min to generate a dark brown precipitate, and it was then washed twice with double-distilled water to remove the lemon extract. The powder precipitate was then dried to produce Ag@SiO_2_@Fe_3_O_4_ NPs for characterization testing. The schematic diagram of the coating process is shown in Figure 2.

### 2.5 Characterization

Using powder X-ray diffraction (XRD, Rigaku D/max-2500), the crystal structures of the produced NPs were examined. The 400–4000 cm^–1^ range of Fourier transform infrared spectroscopy (FTIR) data was collected to understand the coating of silica and silver on the surface of magnetite NPs. The morphologies of the as-prepared products were characterized by transmission electron microscopy (TEM, JEOL JEM-2100, Japan) at 200 kV and field-emission scanning electron microscopy (FESEM, JSM-6700F, JEOL, Japan). The FESEM-integrated energy-dispersive X-ray spectrometry (EDS) examined the chemical composition. Dynamic light scattering (DLS, Zeta sizer Nano ZS-90, Malvern, U.K.) was utilized to confirm the hydrodynamic size and zeta potential. Using a vibrating sample magnetometer (VSM, Lake Shore 7400) and an external magnetic field varying from 15 kOe to 15 kOe, the magnetic characteristics of the produced NPs were determined. The NPs’ surface area was evaluated using a Brunauer–Emmette–Teller (BET) system (Micromeritics ASAP 2020, USA).

### 2.6 Determination of Antibacterial Activity

The antibacterial ability of Fe_3_O_4_, SiO_2_@Fe_3_O_4_, and Ag@SiO_2_@Fe_3_O_4_ NPs was evaluated by using Kirby–Bauer disk diffusion and microdilution techniques of the National Committee of Clinical Laboratory Standards. In brief, bacterial suspension was spread on the Mueller Hinton Agar (MHA) plates, and then impregnated paper discs by NPs were placed on the MHA plates. After incubation for 24 h at 37 °C, the results were determined by measuring the diameter of the zone of inhibition.^38^ Minimum inhibitory concentration (MIC), the lowest concentration of an antimicrobial agent that inhibits visible bacterial growth, and minimum bactericidal concentration (MBC), the lowest concentration of an antimicrobial agent that kills 99.99% of a particular microorganism, were evaluated.^39^*Escherichia coli* (*E. coli*), a Gram-negative bacterium, and *Staphylococcus aureus* (*S. aureus*), a Gram-positive bacterium, were selected as indicators.^40,41^ All culture vessels, tubes, and supplies were autoclave-sterilized prior to usage. 10 μL of bacterial suspension was added to each well of 96-well microtiter plates containing 900 μL of the respective liquid nutrients. Following that, 90 μL of serially diluted concentrations of synthesized Fe_3_O_4_, SiO_2_@Fe_3_O_4_, and Ag@SiO_2_@Fe_3_O_4_ NPs (ranging from 1000 to 10 μg/mL) were added to each well. As a positive control, bacteria without NPs have been used, and the plates were incubated at 37 °C for 24 h. The quantity of bacterial growth was then determined by measuring the optical density at 600 nm using an enzyme-linked immunosorbent assay (ELISA) microtiter plate reader. This procedure was repeated three times. The growth inhibition percentage (GI percent) of NPs was established using the following formula:
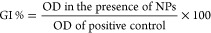


To examine the reusability, Ag@SiO_2_@Fe_3_O_4_ NPs were collected by an external magnet, dispersed in distilled water, and mixed with fresh bacterial suspension for the next antimicrobial cycle.

### 2.7 Cytotoxicity Effects

Human normal skin cell lines (HSF 1184) (5 × 10^4^ cells/well) were seeded on a 96-multiwell dish containing RPMI 1640 medium (supplemented with 10% heat-inactivated fetal bovine serum (FBS) and 1% streptomycin–penicillin) and incubated for 24 h. Then, cell suspensions were incubated with Ag@SiO_2_@Fe_3_O_4_ NPs at different concentrations (1 and 10 μg/mL) in a humidified atmosphere of 5% CO_2_, 95% air at 37 °C. Next, 20 μL of MTT (thiazolyl blue tetrazolium bromide) dye (5 mg/mL in phosphate-buffered saline) was added to each well and incubated for 4 h. Later, 200 μL of dimethyl sulfoxide (DMSO) solution was added, and after 15 min, the absorption was recorded at a wavelength of 570 nm using an ELISA microtiter plate reader (enzyme-linked immunosorbent assay).^17^

## 3 Results and Discussion

**Figure 3:**
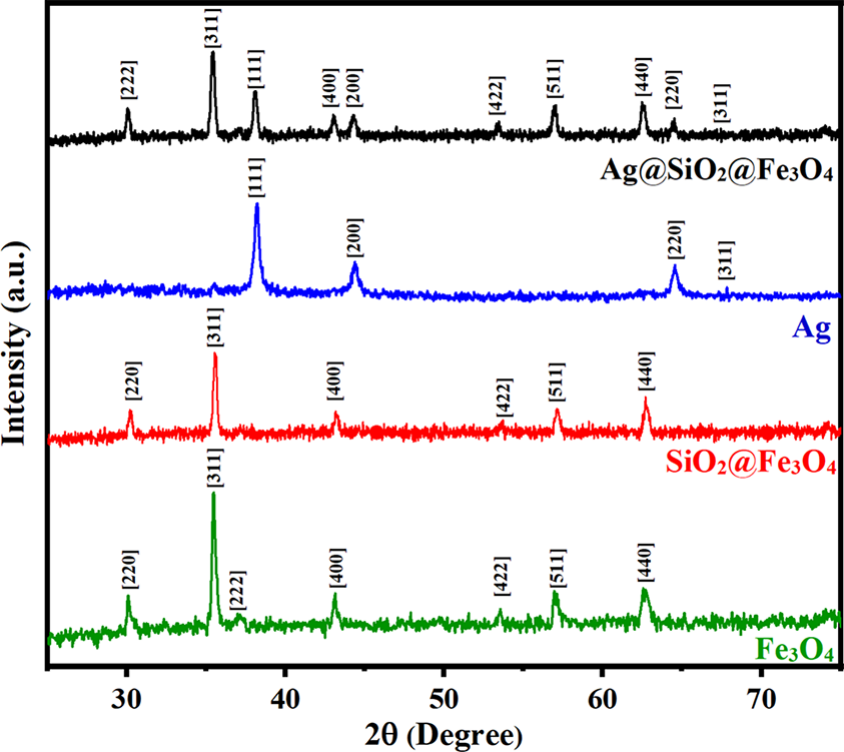
XRD pattern of Fe_3_O_4_, SiO_2_@Fe_3_O_4_, Ag, and Ag@SiO_2_@Fe_3_O_4_ NPs.

For the pure Fe_3_O_4_, the diffraction peaks associated with Bragg reflections at 2θ∼30°, 35°, 38°, 43°, 58°, and 64° were attributed to the magnetite crystal planes (220), (311), (222), (400), (422), (511), and (440), respectively (JCPDS No: 74-0449) (Figure 3). All samples possessed the identical cubic inverse spinel structure, demonstrating that the alteration had no influence on the crystalline structure of magnetite NPs. The *d* values extracted from the XRD spectra are well indexed to the inverse cubic spinel phase of Fe_3_O_4_. The average crystallite size (*D*) of the particles was around 25 nm based on the Scherrer equation: *D* = (*K*λ)/(βcos θ), where *K* is the Scherrer constant, λ is the X-ray wavelength, β is the peak width of half-maximum, and θ is the Bragg diffraction angle.^17^ The presence of the same distinctive peaks in SiO_2_@Fe_3_O_4_ suggests that the crystalline structure of Fe_3_O_4_ NPs is unaffected by the silica surface modification. However, the peak intensities of Fe_3_O_4_ NPs are lower than those of SiO_2_@Fe_3_O_4_ NPs, which may be due to the shielding effect of the amorphous silica shell.^42^ Because of the generated coating’s amorphous structure, no peaks associated with silicon oxide were seen. The diffracted X-ray amount is reduced by the addition of amorphous silica, resulting in decreased peak intensities. Fe_3_O_4_ NPs are coated with silica due to condensation and hydrolysis reactions, which result in the formation of a silica structure on the surface of the NPs.^43^ After coating with silica, the average crystalline size was computed using the Scherrer equation and found to be 55 nm, which agrees with the FESEM results. The XRD spectrum of Ag reveals four distinct diffraction peaks centered at 2θ values of ∼38°, 40°, 64°, and 69° which are attributed to the (111), (200), (220), and (311) planes (JCPDS No: 04-0783). These peaks, although lower in intensity, were present in the spectra of Ag@SiO_2_@Fe_3_O_4_ NPs, indicating the successful loading of Ag NPs onto the surface of SiO_2_@Fe_3_O_4_ NPs. Using the Scherrer equation, the average size of the Ag crystallites in Ag@SiO_2_@Fe_3_O_4_ NPs was demonstrated to be ∼90 nm, which was in good agreement with the average particle size acquired from TEM examination.

Table 1 summarizes the size of silica and silver-coated magnetite NPs in this work and compares it with other types of chemical synthesis methods. Apparently, green synthesis yields smaller NPs compared to other means of synthesis. Using a green stabilizer, such as citrus lemon extract, can be a viable method for producing NPs. Thus, plant extracts regulate NP synthesis in a single-step, high-yield synthesis to create well-defined sizes and morphologies.^44^

**Table 1:** Sizes of Ag@SiO_2_@Fe_3_O_4_ NPs Produced via Different Synthesis Routes

method	size (nm)	ref
Stöber sol–gel process	400	(31)
microemulsion co-precipitation method	300	(45)
chemical method	280	(46)
sonication method	200	(33)
solvothermal reaction	200	(32)
green synthesis	90	current work

**Figure 4:**
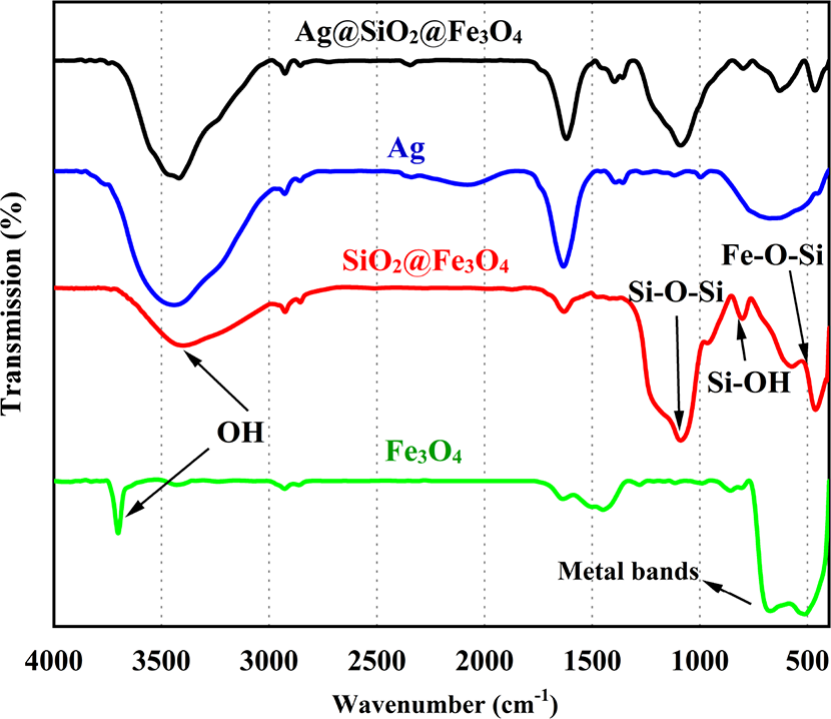
FTIR spectra of Fe_3_O_4_, SiO_2_@Fe_3_O_4_, Ag, and Ag@SiO_2_@ Fe_3_O_4_ NPs.

The FTIR spectra of Fe_3_O_4_, SiO_2_@Fe_3_O_4_, Ag, and Ag@SiO_2_@Fe_3_O_4_ NPs are shown in Figure 4. The stretching vibration of the Fe–O–Fe bond is attributed to the band in the range of 300–600 cm^–1^, and the absorption peaks at around 562 cm^–1^ result from the splitting of the ν_1_ band at 570 and 445 cm^–1^ from the shifting of the *ν*_2_ band, which corresponds to the Fe–O bond of bulk magnetite, also confirming the presence of Fe_3_O_4_.^47^ For the SiO_2_@Fe_3_O_4_ data, the assignments of the bands Si–O–Si (1080 and 791 cm^–1^), Si–OH (945 cm^–1^), and Fe–O–Si (458 cm^–1^) provide evidence that the surface of the magnetic core is coated with silica.^48^ The stretching vibration of OH groups was attributed to the peak with the most characteristic shape at 3415 cm^–1^ that it might be related to the hydroxyl groups and absorbed water in SiO_2_.^49^ Compared to SiO_2_@Fe_3_O_4_, the peaks of the metal bands in the Ag@SiO_2_@Fe_3_O_4_ spectrum shifted from 461 to 468 and 571 to 622 cm^–1^, respectively, indicating that silver NPs were deposited on the SiO_2_@Fe_3_O_4_ NPs.^45^ The FTIR spectra indicated a considerable change in the absorption peaks at 1021, 1443, 1634, and 3428 cm^–1^, indicating that the functional groups interacted with the Ag NPs’ surface. The other small absorption peaks located at 616, 1077, 1355, 1629, 2923, and 3351 cm^–1^ correspond to several functional groups like CO, CH (stretch), C=C, CH (bending), and OH of lemon extract, respectively.

**Figure 5:**
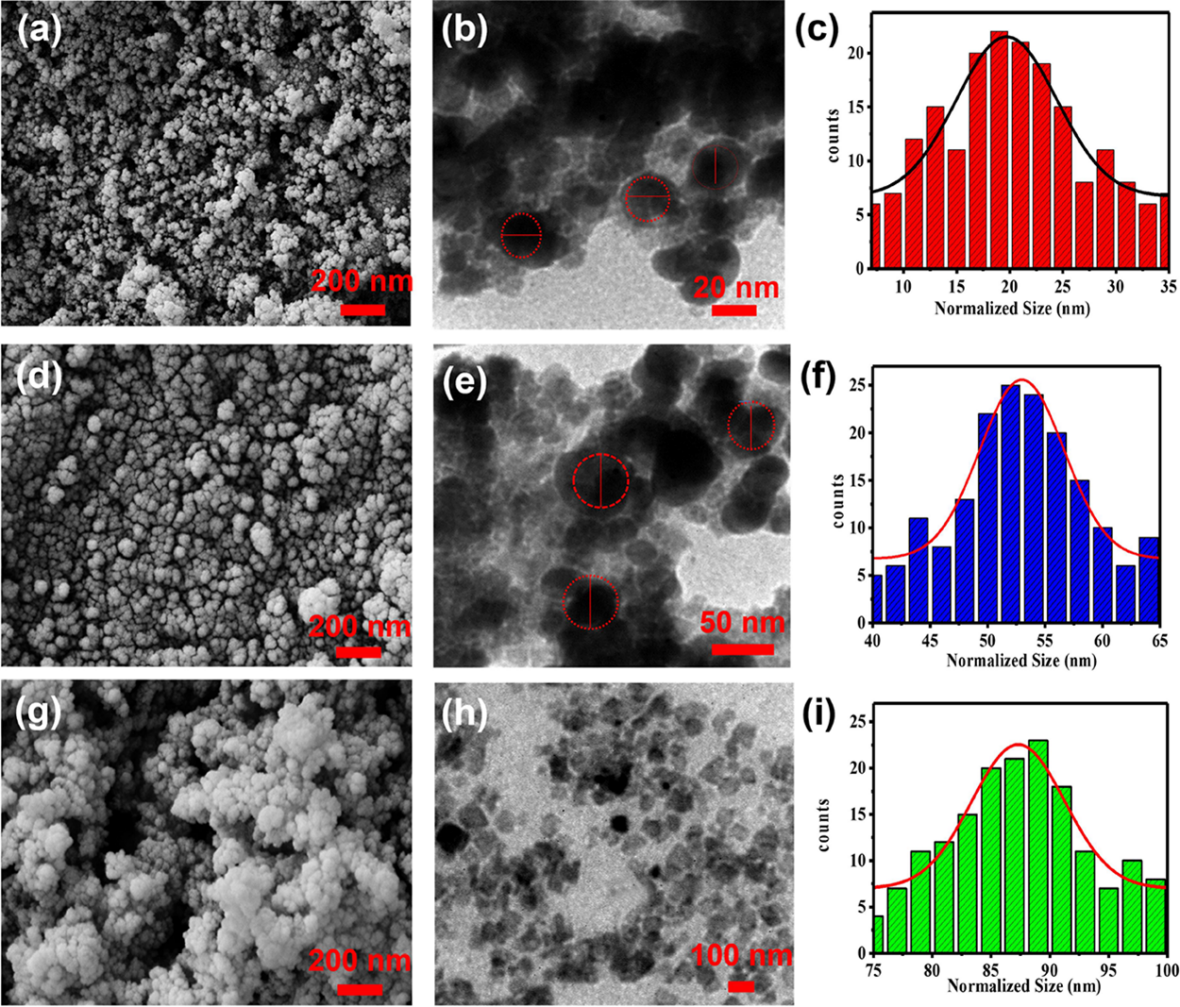
FESEM and TEM images and size distribution histograms of (a–c) Fe_3_O_4_, (d–f) SiO_2_@Fe_3_O_4_, and (g–i) Ag@SiO_2_@Fe_3_O_4_ NPs.

FESEM and TEM images of the magnetite particles without coating show that the average particle size with significant aggregation was in the 20–30 nm range (Figure 5a,b). Bare magnetite NPs easily aggregate, although the morphology of the particles is quite uniform and they exhibit a narrow size distribution (Figure 5c).^50^ It was previously reported that Fe_3_O_4_ NPs have a strong tendency to aggregate, yet they display strong magnetism, high specific surface area, and high surface energy. They disperse poorly in solutions, which makes it hard to coat their surface and deposit shell layers on them.^51^ In order to overcome this challenge and properly deposit SiO_2_ particles on the Fe_3_O_4_ core, several parameters are essential, which include the amount and ratio of water and ethanol, reaction time, amount of Fe_3_O_4_ NPs, concentration of ammonia, ultrasonication, and the use of a mechanical stirrer. Besides, changing the TEOS precursor concentration allows adjustment of the SiO_2_ shell thickness, which is crucial because as the coating gets thicker, magnetic properties diminish. FESEM and TEM images show that the SiO_2_@Fe_3_O_4_ composite with a core/shell structure had a larger size compared to bare magnetite NPs (Figure 5d,e). TEM images showed the thickness of dense silica shell NPs to be ∼20 nm, and the histogram showed that the average size of particles with a narrow size distribution was evaluated as ∼55 nm (Figure 5f). The addition of Ag NPs to SiO_2_@Fe_3_O_4_ resulted in a distinct surface with good size uniformity and an increase in the average size of NPs to ∼90 nm. So, TEM analysis was conducted to learn more about the core/shell structure of Ag@SiO_2_@Fe_3_O_4_ NPs. Due to the small size of the silver NPs and their resemblance to the core/shell nanostructure, the silver coating on silica is completely compacted, and the size of Ag NPs was calculated to be approximately 30 nm. Silver NPs with small sizes (∼20 nm) tend to aggregate, which limits their practical applications, such as reduced antibacterial effectiveness. The NP aggregations are clearly visible in Figure 5a,b. To solve this problem, silica coating was employed to support silver NPs, and the ultrafine silver NPs can be homogeneously formed on the surface of SiO_2_@Fe_3_O_4_ with less aggregation (Figure 5h). After coating with silica, silver aggregation was decreased, so NPs could easily attach to bacterial cell walls, which is a key factor in their growth inhibition.^52^

**Figure 6:**
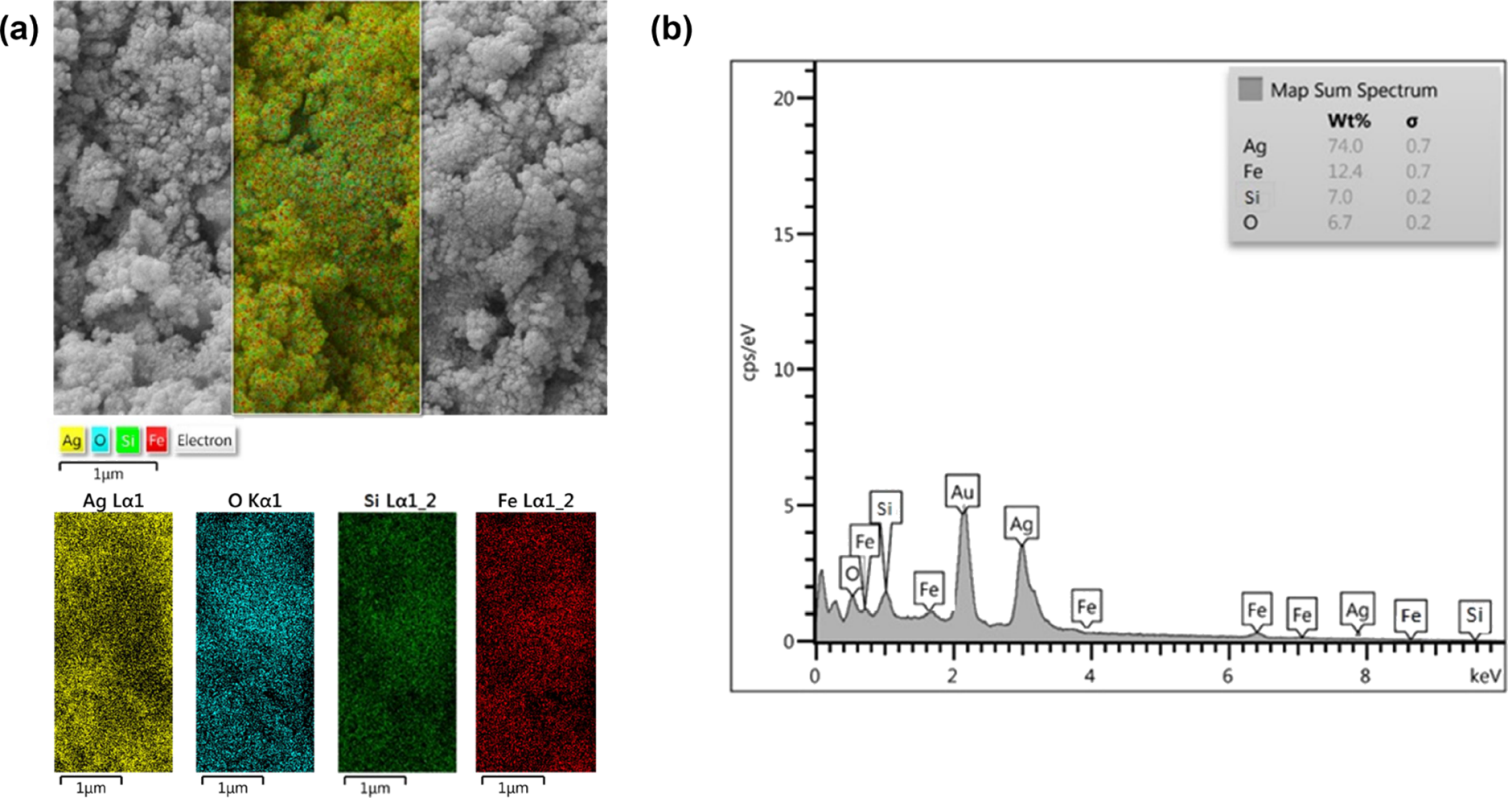
(a) Elemental mapping and (b) EDS analysis of Ag@SiO_2_@Fe_3_O_4_ NPs.

To confirm the successful preparation of Ag@SiO_2_@Fe_3_O_4_ NPs, the samples were analyzed with electron mapping (Figure 6a). Fe and O were found to be distributed more homogeneously for all the examined particles, and Fe is detected inside these particles whereas Si and Ag are found on the exterior of Fe_3_O_4_ NPs. These findings show that the Ag NPs and SiO_2_ nanolayers are uniformly spread across the surface of Fe_3_O_4_, although this is difficult to discern for extremely tiny particles.^53^ The identification of O, Si, Fe, and Ag elements in Ag@SiO_2_@Fe_3_O_4_ NPs by EDS analysis confirmed the existence of the coating of Ag and SiO_2_ on the Fe_3_O_4_ NPs (Figure 6b).^49^ The Au peak is due to the sample preparation, where a thin layer of gold was coated.

**Figure 7:**
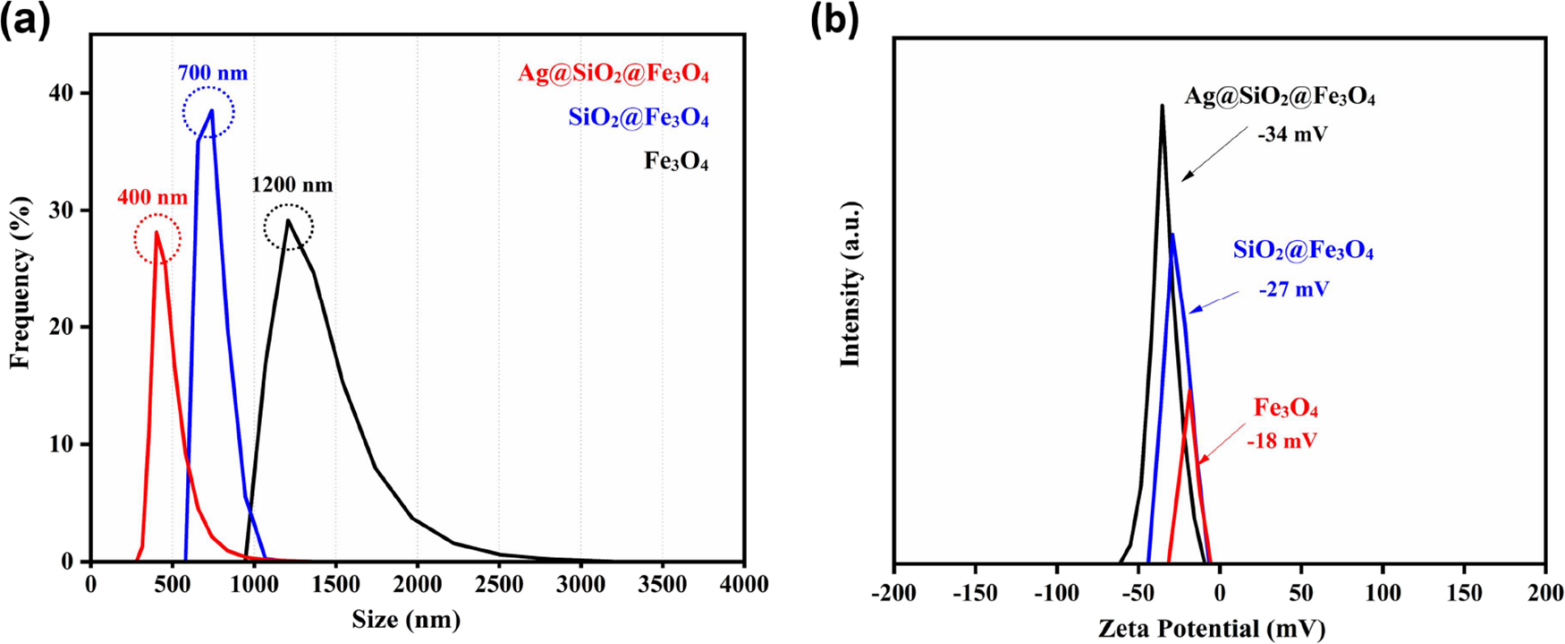
(a) Size distribution and (b) zeta potential measurements of Fe_3_O_4_, SiO_2_@Fe_3_O_4_, and Ag@SiO_2_@Fe_3_O_4_ NPs.

To further characterize the colloidal behavior of NPs in aqueous media, DLS analysis was performed. Bare and coated NPs (0.1 g) in distilled water were used to obtain hydrodynamic size and size distribution information. The average hydrodynamic diameter of Fe_3_O_4_ NPs was ∼1200 nm, which demonstrates the influence of agglomeration of bare magnetite NPs. SiO_2_@Fe_3_O_4_ and Ag@SiO_2_@Fe_3_O_4_ NPs had a mean hydrodynamic diameter of ∼700 and ∼400 nm, respectively (Figure 7a). The capping effect of silica and the addition of silver NPs clearly resulted in a decrease in measured hydrodynamic size values, indicating increased stability. Zeta potentials for bare, silica-coated, and final NPs were −18, −27, and −34 mV, respectively. Silica coating led to a decrease in surface charge due to the silanol groups of the silica, and silver, as a diamagnetic material, decreases the direct magnetic interactions between the magnetite NPs, hence reducing their agglomeration. Therefore, coating magnetite NPs with silver and silica enhanced their colloidal stability. Elsewhere, Fe_3_O_4_ and Fe_3_O_4_@SiO_2_ NPs were synthesized by the microemulsion method, and the mean diameters of the NPs decreased from 1765 to 459 nm due to the silica coating, similar to the behavior observed in this study.^54^Table 2 summarizes the results for hydrodynamic size and zeta potential measurements.

**Table 2:** Zeta Potential, Hydrodynamic Diameter (Mean Diameter), and Polydispersity Index of Fe_3_O_4_, SiO_2_@Fe_3_O_4_, and Ag@SiO_2_@Fe_3_O_4_ NPs^a^

sample	zeta potential (mV)	hydrodynamic diameter (nm)	polydispersity index (PDI)
Fe_3_O_4_	–18	1200	0.215
SiO_2_@Fe_3_O_4_	–27	700	0.173
Ag@SiO_2_@Fe_3_O_4_	–34	400	0.156

a Polydispersity index values of around 0.200 indicate the monodispersity.

**Figure 8:**
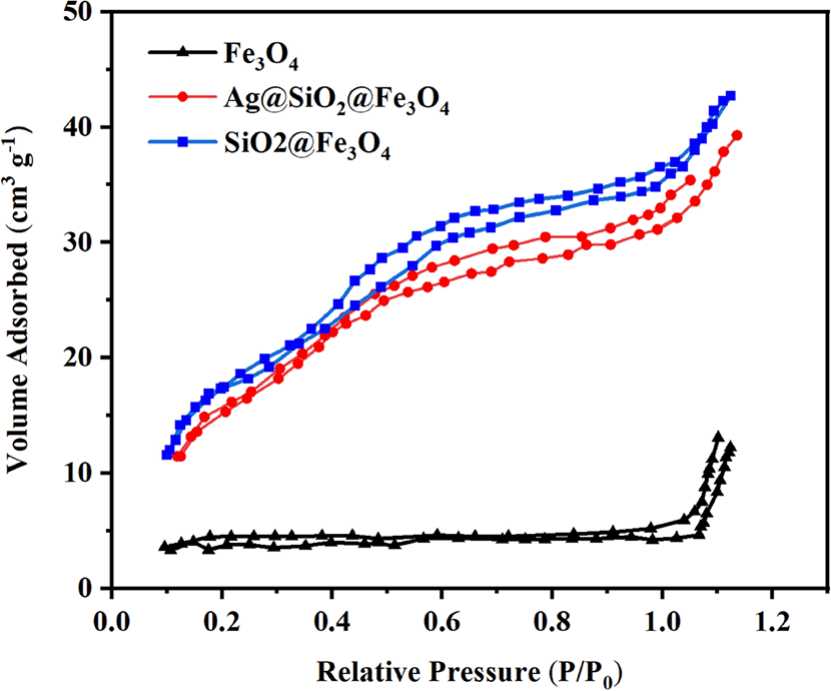
N_2_ adsorption–desorption isotherms of Fe_3_O_4_, SiO_2_@Fe_3_O_4_, and Ag@SiO_2_@Fe_3_O_4_ NPs.

The specific surface area (*S*) of each dry sample (0.02 g) was assessed by the N_2_ sorption/desorption (BET) technique, and the nitrogen adsorption–desorption isotherms were used for the calculation of the surface areas of the synthesized materials (Figure 8). The maximum specific surface areas of bare, silica-coated, and final NPs were 67, 180, and 98 m^2^ g^–1^, respectively. SiO_2_-coated NPs have multiple nanopores in their walls; hence, coating with silica substantially increased the NPs’ surface area. For Ag@SiO_2_@Fe_3_O_4_ NPs, the effect is described by the combined influence of the increase in particle diameter and decreased particle density, where both parameters have opposite effects on the resultant specific surface area value.^55^ This observation implies that silver did not completely cover the SiO_2_@Fe_3_O_4_ NPs, and on the surface of SiO_2_@Fe_3_O_4_, silver NPs are likely to organize into ″island-like″ formations. Such surface layer organization will increase the surface area of Ag@SiO_2_@Fe_3_O_4_ NPs in contact with bacteria or viruses, significantly enhancing the bactericidal activity. Particles with a high specific surface area are known to have a high level of chemical and biological activity.^56^

**Figure 9:**
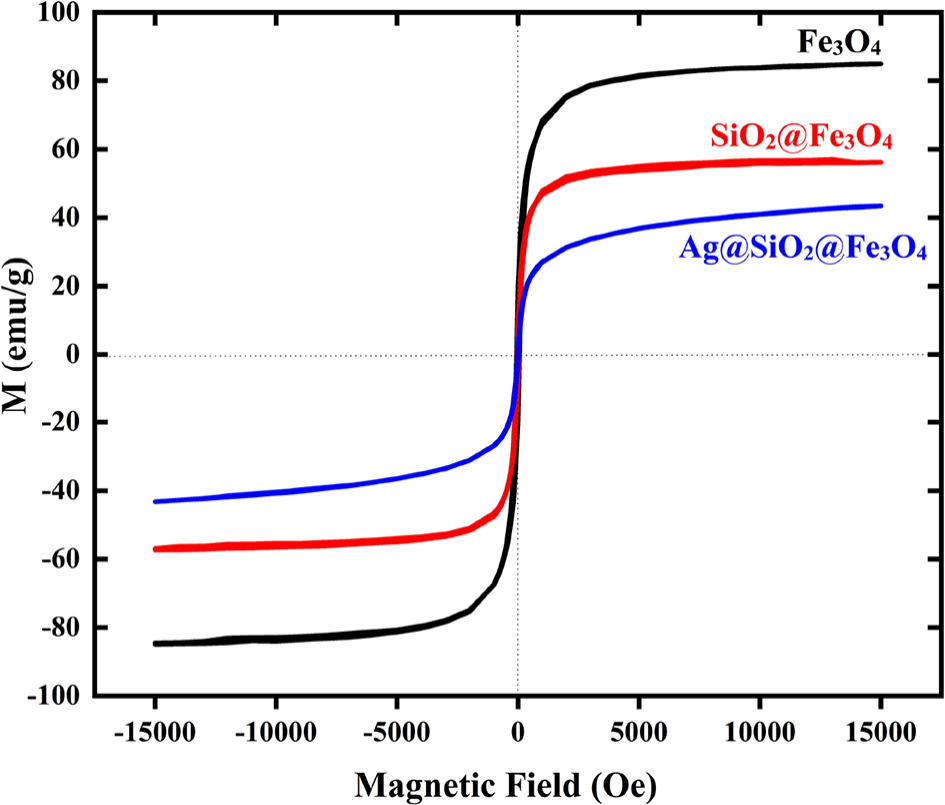
Hysteresis loops of Fe_3_O_4_, SiO_2_@Fe_3_O_4_, and Ag@SiO_2_@Fe_3_O_4_ NPs.

The magnetic characteristics of the NPs were evaluated using a superconducting quantum interference device (SQUID) magnetometer at 300 K.^57^ The saturation magnetization (*Ms*) values of the bare Fe_3_O_4_, SiO_2_@Fe_3_O_4_, and Ag@SiO_2_@Fe_3_O_4_ NPs were found to be 78.5, 56.4, and 43.8 emu/g, respectively (Figure 9). Due to the ultrafine magnetite nanocrystal composition, they displayed superparamagnetic properties and had low remanence and coercivity.^45^ The immobilization of amorphous silica and adhesion of Ag NPs caused the magnetic saturation of Fe_3_O_4_ NPs to decrease. Ag@SiO_2_@Fe_3_O_4_ NPs had a slightly lower *M*_s_ value than SiO_2_@Fe_3_O_4_ microspheres, which is explained by the slight increase in mass and size caused by the deposition of Ag NPs.^32,33^ Nonetheless, the bifunctional NPs displayed considerable magnetism, indicating their usefulness for magnetic separation and targeting. Therefore, through green synthesis, superparamagnetic NPs with enhanced magnetization were produced.

**Figure 10:**
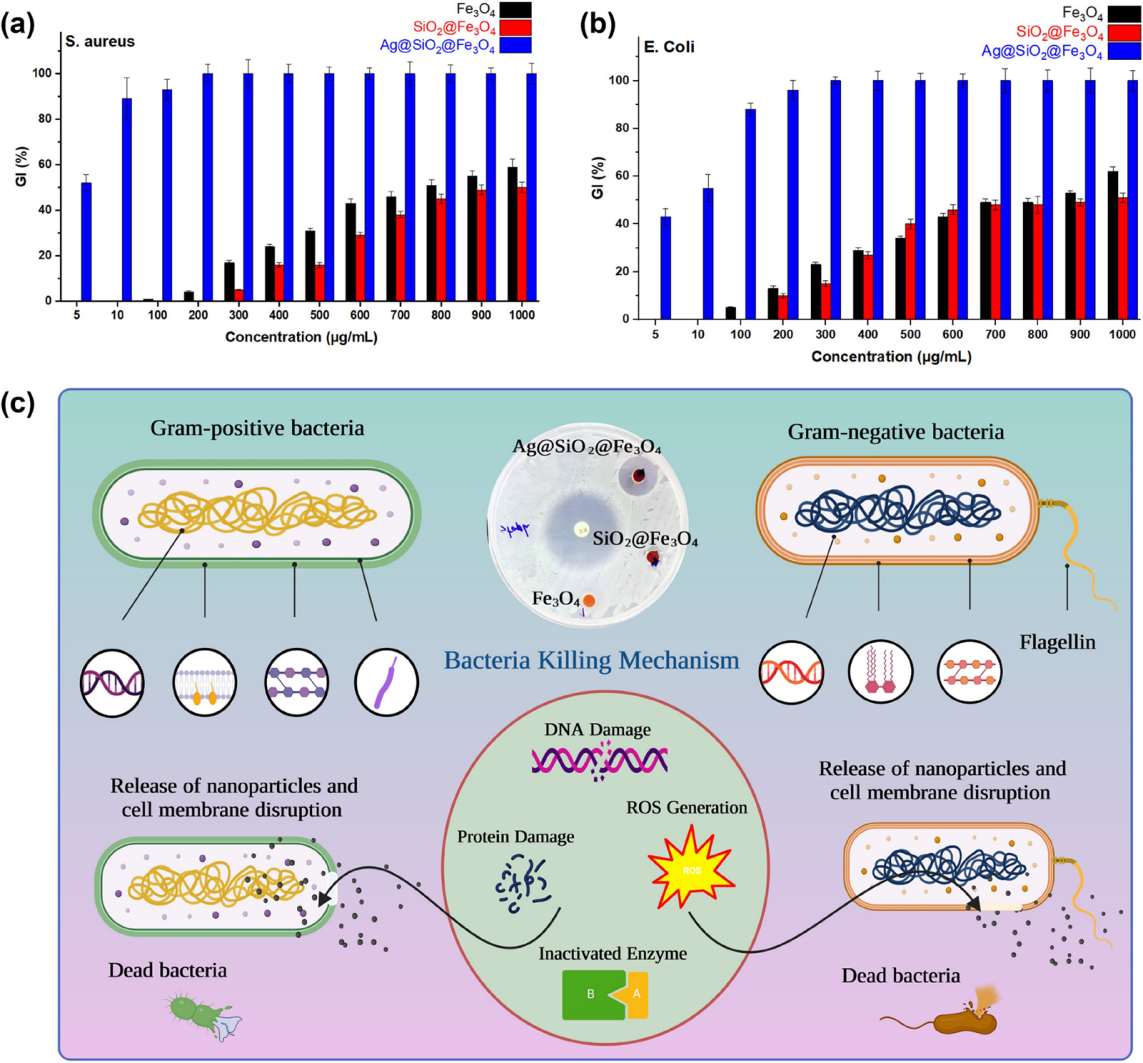
Effect of different concentrations (μg/mL) of Fe_3_O_4_, SiO_2_@Fe_3_O_4_, and Ag@SiO_2_@Fe_3_O_4_ NPs against (a) *S. aureus* and (b) *E. coli*. (c) Schematic diagram of the antibacterial activity mechanism of the NPs on Gram-positive and Gram-negative bacteria.

To demonstrate the bacteria inhibition performance of Fe_3_O_4_, SiO_2_@Fe_3_O_4_, and Ag@SiO_2_@Fe_3_O_4_ NPs, *S. aureus* and *E. coli* were used as indicators, and broth microdilution tests were performed. The bacterial growth inhibition increases with increasing NP concentration (Figure 10a,b). At a concentration of 5 μg/mL, Ag@SiO_2_@Fe_3_O_4_ NPs showed excellent inhibitory efficiency compared to Fe_3_O_4_ and SiO_2_@Fe_3_O_4_, resulting in 52 and 43% of the growth inhibition of *S. aureus* and *E. coli* bacteria, respectively. Fe_3_O_4_ NPs and SiO_2_@Fe_3_O_4_ NPs did not have sufficient inhibitory activity until reaching the concentrations of 1000 μg/mL. Therefore, the higher antibacterial effect of Ag@SiO_2_@Fe_3_O_4_ NPs can be explained by the incorporation of silver atoms in the structure.

Table 3 shows the MIC and MBC values, demonstrating that Ag@SiO_2_@Fe_3_O_4_ NPs had the lowest MIC and MBC values against *S. aureus* and *E. coli* compared to Fe_3_O_4_ and SiO_2_@Fe_3_O_4_ NPs. According to the disc diffusion results, higher antibacterial activity was observed in the presence of Ag@SiO_2_@Fe_3_O_4_ NPs against *S. aureus* with an inhibition zone of 23 mm, and these results confirmed the MIC and MBC outcomes (Table 3).

**Table 3:** Zone of Inhibition (mm) and MIC and MBC Data of Fe_3_O_4_, SiO_2_@Fe_3_O_4_, and Ag@SiO_2_@Fe_3_O_4_ (in μg/mL) for Gram-Negative and Gram-Positive Bacteria

bacterial strains	Fe_3_O_4_ (μg/mL)			SiO_2_@Fe_3_O_4_ (μg/mL)			Ag@SiO_2_@Fe_3_O_4_ (μg/mL)		
	zone of inhibition (mm)	MIC	MBC	zone of inhibition (mm)	MIC	MBC	zone of inhibition (mm)	MIC	MBC
*S. aureus*	∼ 0	800	>1000	∼ 0	1000	>1000	∼23	5	200
*E. coli*	∼ 0	900	>1000	∼ 0	1000	>1000	∼20	10	300

The inhibitory experiments showed that Ag@SiO_2_@Fe_3_O_4_ NPs inhibited *S. aureus* growth more efficiently than *E. coli*. Generally, the amount of interaction between bacteria and NPs depends on the peptidoglycan thickness of the bacterial membrane. Gram-positive bacteria have a single, thick cell wall that is composed of peptidoglycan and teichoic acids. Ag@SiO_2_@Fe_3_O_4_ NPs may adhere to *S. aureus* more firmly and rapidly than to *E. coli*. Nevertheless, as a Gram-negative bacterium, *E. coli* possesses a single peptidoglycan layer. Despite having a significantly thicker cell wall than *E. coli*, *S. aureus* was extremely sensitive to Ag@SiO_2_@Fe_3_O_4_ NPs at low concentrations. The difference in sensitivity can be explained by the number of membranes that each microorganism has. *E. coli* has two cell membranes, compared to *S. aureus*, which has only one. Additionally, the periplasm and outer membrane of *E. coli* increase its resistance to Ag@SiO_2_@Fe_3_O_4_ NPs.^58^ The bactericidal mechanism of Ag@SiO_2_@Fe_3_O_4_ NPs is not fully understood. Recent studies have advised that when *S. aureus* is treated with NPs, its membrane morphology changes.^59^ Due to the presence of functional groups on the surface of bacterial cell walls, such as carboxyl, hydroxyl, and phosphate, the total charge of bacteria at biological pH values is negative.^60^ However, electrostatic interaction between positively charged NPs and negatively charged bacteria can improve the antibacterial efficacy of particles, but it is not the only factor that can influence biocompatibility and bactericidal properties. The high antibacterial efficiency of the negatively charged Ag@SiO_2_@Fe_3_O_4_ NPs synthesized in the present study has been attributed to a combination of their distinctive characteristics, including NP size, stability, and surface chemistry.^61^ Due to their large surface area and reactivity, smaller particles, for instance, were revealed to have a significant antibacterial effect.^62^ It is probable that the antibacterial activity of Ag@SiO_2_@Fe_3_O_4_ NPs is due to both the interaction of released Ag^+^ with the functional groups of vital enzymes and proteins and the collapsing force generated by reactive oxygen species (ROS) (Figure 10c).^63^ Other studies have suggested that the presence of silver on the surface of bacterial cells causes cell membrane rupture or renders osmosis inactive due to ROS, destroying proteins and DNA in bacteria.^64^ Because of their unique and widespread bactericidal function, Ag NPs have been extensively exploited for killing practically all species of microorganisms.

Kittler et al. reported that bacterial growth was inhibited by bare Ag NPs at concentrations above 108 μg/mL.^65^ However, Ag NPs in contact with microbial cells in medium culture tend to agglomerate, which drastically reduces their antibacterial effect. This may also drive the usage of additional Ag NPs, which may potentially have environmentally harmful effects. The strong inhibitory ability of Ag@SiO_2_@Fe_3_O_4_ NPs is due to the cooperative action of Ag and Fe_3_O_4_, as well as SiO_2_@Fe_3_O_4_ serving as a stabilizer to minimize the aggregation of Ag NPs. Lemon extract, which was used to synthesize Ag@SiO_2_@Fe_3_O_4_ NPs, also exhibits mild inhibitory action.^66^ It was recently shown that immobilizing NPs can increase their antibacterial capabilities, which supports the results of the current work.^67^

The reusability of the NPs is a crucial property that needs to be considered in practical applications. Ag@SiO_2_@Fe_3_O_4_ NPs were recovered after usage via magnetic separation, which was repeated twelve times. According to the MIC and MBC values of the Ag@SiO_2_@Fe_3_O_4_ NPs, the initial concentration for reusability was chosen as 200 μg/mL.^68^ The results of bacterial growth inhibition in twelve repeated cycles were tested. After 11 cycles, the antibacterial capacity of Ag@SiO_2_@Fe_3_O_4_ NPs was around 98%, which shows that the novel nanocomposite synthesized in the current work can be successfully recycled and reused in antimicrobial applications.

**Figure 11:**
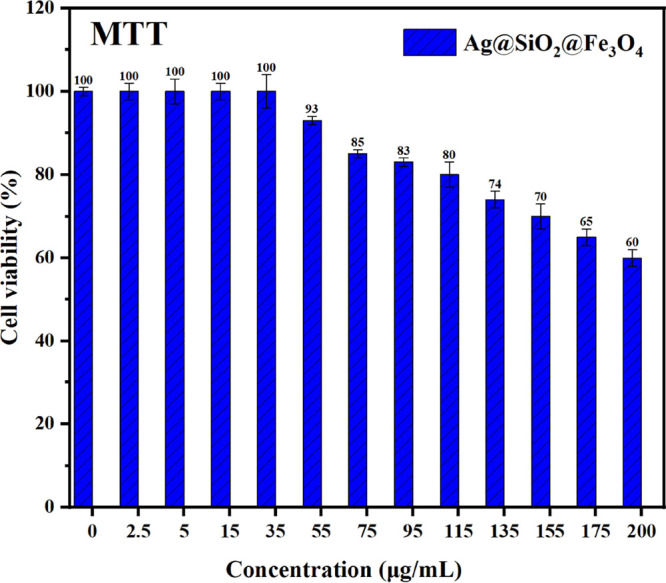
Percentage of cell viability of HSF 1184 cells in the presence of different concentrations of Ag@SiO_2_@Fve_3_O_4_ NPs.

The toxicology of nanocomposites greatly restricts their medical, cosmetic, and environmental applications. The antibacterial performance of Fe_3_O_4_ and SiO_2_@Fe_3_O_4_ was poor; therefore, their cell viability tests were not performed in the current work, while previous reports demonstrated that these NPs had low cytotoxicity even at high concentrations.^69,70^ Small-sized silver and Fe_3_O_4_ NPs tend to aggregate to decrease surface energy during preparation, resulting in a significant loss of their antimicrobial capabilities. To address this problem, silica is an ideal support material for effectively loading NPs as electrostatic interactions allow silver cations to form direct bonds with the functional groups on the silica surface, and the aggregation issue can be addressed by using silica as the support material.^71,72^ We studied how the cell viability of normal human skin exposed to Ag@SiO_2_@Fe_3_O_4_ NPs was affected. There was no obvious cytotoxicity of Ag@SiO_2_@Fe_3_O_4_ NPs at their MIC or MBC dose (Figure 11). As reported in previous studies, Ag NPs exhibited serious toxicity even at lower concentrations,^28^ and cationic NPs are more cytotoxic than those with neutral or negative surface charges.^73^ Therefore, as a further advancement, synthesized Ag@SiO_2_@Fe_3_O_4_ NPs, due to their negative surface charge, can confer on these NPs an optimal alternative to cosmetic and food preservatives.

## 4 Conclusions

This work demonstrated a successful preparation, excellent antibacterial performance, low cytotoxicity, and reuse via magnetic recovery of Ag@SiO_2_@Fe_3_O_4_ nanocomposite particles produced by a green synthesis method using lemon fruit extract. Various methods were used to evaluate the NPs, and their antibacterial efficiency was tested against Gram-positive and Gram-negative bacteria strains. Antibacterial mechanisms can be explained by the release of Ag ions and the strong oxidation of ROS. It has the additional benefit of being easily extracted from water using a magnetic field to prevent environmental pollution. So, Ag@SiO_2_@Fe_3_O_4_ nanoparticles hold great promise as a novel, effective, biocompatible, and reusable antibacterial agent due to their good reproducible antibacterial efficacy during subsequent recycling.

## Data Availability

The authors confirm the absence of sharing data.
